# A New, MWCNT-Based, Solid-State Thiabendazole-Selective Sensor

**DOI:** 10.3390/s22103785

**Published:** 2022-05-16

**Authors:** Andrea Dandić, Ivana Novak, Marija Jozanović, Iva Pukleš, Aleksandar Széchenyi, Mateja Budetić, Mirela Samardžić

**Affiliations:** 1 Department of Chemistry, Josip Juraj Strossmayer University of Osijek, Cara Hadrijana 8/A, 31000 Osijek, Croatia; andreajuric@kemija.unios.hr (A.D.); ivana.novak@kemija.unios.hr (I.N.); mjozanovic@kemija.unios.hr (M.J.); szealex@kemija.unios.hr (A.S.); 2 Doctoral School of Chemistry, University of Pécs, Ifjúság útja, 7624 Pécs, Hungary; jcf9xs@pte.hu

**Keywords:** thiabendazole, solid-state sensor, direct potentiometry

## Abstract

Direct potentiometric measurements using solid-state sensors have a great potential for thiabendazole (TBZ) determination, considering simplicity, accuracy, and low cost. Modifying the sensing material of the sensor with multi-walled carbon nanotubes (MWCNTs) leads to improved analytical properties of the sensor. In this study, a new potentiometric solid-state sensor for TBZ determination, based on MWCNTs modified with a sulfate group, and TBZ ion as sensing material was developed. The sensor exhibited a Nernstian response for TBZ (60.4 mV/decade of activity) in a working range between 8.6 × 10^−7^ and 1.0 × 10^−3^ M. The detection limit for TBZ was 6.2 × 10^−7^ M. The response time of the sensor for TBZ was 8 s, and its signal drift was only 1.7 mV/h. The new sensor is applicable for direct potentiometric determination of TBZ in complex real samples, such as fruit peel. The accuracy of TBZ determination is confirmed using the standard addition method.

## 1. Introduction

Thiabendazole (TBZ) is a benzimidazole derivative with a wide area of application as a fungicide. It is one of the most detected pesticides in the United States of America and Europe [1]. TBZ is used for fruit and vegetable treatment to prevent mold, pests, and rot. It is highly used in bananas and citrus fruit to keep freshness and as a component of the waxes applied on the fruit, respectively [2,3]. In citrus fruits and bananas, the maximum residue levels of TBZ, permitted by the European food safety authority, are 7 and 6 mg/kg, respectively [4]. Mainly, TBZ is localized in the peel of the fruit. Although it has low acute toxicity, the US Environmental Protection Agency (EPA) has classified TBZ as likely to be carcinogenic at doses high—enough to cause disturbance of thyroid hormone balance [3]. However, a diet that includes crops treated with TBZ (according to guidelines for safe use of crop protection products) will result in much lower concentrations of TBZ in the human body than those sufficient to cause cancer. In addition, TBZ causes liver toxicity [5] and nephrotoxicity [6]. It is declared as a food additive in the European Union as E233 [7,8]. The allowable daily intake of TBZ is 0.1 and 0.3 mg/kg according to EPA and World Health Organization, respectively, and the no observed effect level for clinical symptoms and blood chemistry changes is 3.3 mg/kg per day for single-dose exposure [9]. Due to the possibility of exposure to TBZ through the diet and its stability during various food processing procedures, it is necessary to monitor TBZ residues in food [10]. Various analytical methods have been used to provide qualitative and quantitative information about TBZ and to ensure food safety.

Chromatographic methods are the most commonly used for TBZ determination [11,12,13,14,15], but other analytical techniques such as surface-enhanced Raman spectroscopy [16,17], UV/Vis spectrophotometry [18], room-temperature phosphorimetry [19], immunoassay [10], fluorimetry [20] and electroanalytical methods [21] are also applicable. However, many of these methods demand high-cost equipment, large consumption of organic solvents, and complicated procedures and sample preparations. Considering their simplicity and good analytical performances, ion-selective electrodes (ISEs) as potentiometric sensors could be a good alternative to these methods. Usually, they have a liquid membrane containing sensing material, plasticizer, and polyvinyl chloride [22]. The sensing properties of ISEs can be optimized by appropriate choices of ionophore and plasticizer and their relative amounts. The main disadvantage of these sensors is the leaching of the sensor material, which leads to changed characteristics and shortened lifetime of the sensor. It could be overcome by using solid-state sensors or by modifying the membrane composition using less water-soluble sensing materials [23]. For that purpose, carbon-based nanomaterials, such as multi-walled carbon nanotubes (MWCNTs), proved to be suitable; they can cause better response characteristics [24], reduce leaching of the sensing material from the membrane, and reduce noise [25]. In addition, they can be covalently modified while retaining their individual properties, resulting in the formation of a hybrid sensing material [26,27].

This paper describes the preparation of a new hybrid material based on MWCNTs modified with a sulfate group and TBZ ion and its application as a sensing material in the liquid membrane for a new solid-state sensor for TBZ determination. This research aimed to develop a simple, fast, accurate, and improved potentiometric method for TBZ determination, which was possible due to introducing modified MWCNTs as a sensor material. A newly developed potentiometric sensor was characterized, and its applicability was demonstrated in pure TBZ solutions and fruit samples.

## 2. Materials and Methods

### 2.1. Sensor Preparation

MWCNT-OSO_3_H (prepared according to [25], using MWCNT-OH (95+%, IoLiTec, Heilbronn, Germany, o.d. of 20 to 30 nm and a length of 10 to 30 μm), chlorosulfonic acid (Acros Organics B.V.B.A., Geel, Belgium), potassium carbonate (K_2_CO_3_, GRAM-MOL, Zagreb, Croatia), and *n*-hexane (Carlo Erba, Cornaredo, Italy)) and nitrate salt of TBZ (prepared according to [28] using TBZ (Acros Organics, Geel, Belgium), Fe(NO_3_)_3_ 9H_2_O (Fluka, Buchs, Switzerland), and absolute ethanol (Fluka, Buchs, Switzerland)) were used for the new sensing material (MWCNT-OSO_3_^−^TBZ^+^) preparation. Then, 100 mg of MWCNT-OSO_3_H was mixed with 2 mL of water and placed in an ultrasonic bath to form a stable homogenous suspension. A TBZ solution (*c* = 1 × 10^−2^ M) was added dropwise in suspension until a flaky precipitate was formed, and it was stirred using a magnetic stirrer for 24 h. The precipitate was washed with water and centrifuged (5 min at 5000 rpm) three times, followed by drying for 24 h at 60 °C. The newly prepared MWCNT-OSO_3_^−^TBZ^+^ (0.0018 g), tetrahydrofuran (THF, Fisher Scientific, Loughborough, UK, 1 mL), dimethylformamide (DMF, GRAM-MOL, Zagreb, Croatia, 10 µL), poly(vinyl chloride) (PVC, Fluka, Buchs, Switzerland, 0.0300 g), and dibutyl sebacate (DS, Fluka, Buchs, Switzerland, 63.94 µL) were mixed in order to prepare the liquid membrane of the sensor. The assembling of the new sensor and drop-casting of graphene layer (Gwent group, Pontypool, UK) and membrane mixture on the graphite core of the sensor were performed according to [25].

### 2.2. Reagents and Materials for Measurements

HCl (Carlo Erba, Cornaredo, Italy) was used to prepare the analyte (TBZ). All salt solutions for interference measurements, L(+)-ascorbic acid (GRAM-MOL, Zagreb, Croatia), citric acid (GRAM-MOL, Zagreb, Croatia), glucose (Kemika, Zagreb, Croatia), fructose (Kemika, Zagreb, Croatia), imazalil (Sigma Aldrich, St. Louis, MO, USA), fuberidazole (Dr. Ehrenstorfer, Augsburg, Germany), carbaryl (Dr. Ehrenstorfer, Augsburg, Germany), carbendazim (Dr. Ehrenstorfer, Augsburg, Germany), 2-phenylphenol (Sigma Aldrich, St. Louis, MO, USA) and NaOH (GRAM-MOL, Zagreb, Croatia) were prepared using analytical grade chemicals. Deionized water (conductivity of 0.055 µS/cm) was used to prepare all solutions.

Bananas, oranges, lemons, clementines, and limes were purchased from the local store in Osijek (Croatia) and were not declared bio fruit. Bananas originated from Ecuador, oranges and clementines from Greece, and lemons and limes from Spain.

### 2.3. Apparatus

An ultrasonic bath (BANDELIN RK-100, Berlin, Germany) was used to prepare the sensor membrane and TBZ solution. Measurements were performed using 794 Basic Titrino coupled with 806 Exchange unit, 826 mobile pH meter, 728 stirrer, Tiamo software (all from Metrohm, Herisau, Switzerland), and in-house software. SEM images of the electrode surfaces were taken by SEM (JEOL JSM-IT500HR, Tokyo, Japan) using back-scattered electron detector. Samples were analyzed without gold coating.

### 2.4. Procedure

All measurements were performed at room temperature with magnetic stirring and without ionic strength adjustment, using a newly developed MWCNT-OSO_3_^−^TBZ^+^ sensor as the working electrode and silver/silver chloride electrode (Metrohm, Herisau, Switzerland) as the reference electrode. Conditioning the sensor in TBZ solution (*c* = 2.0 × 10^−3^ M) for 15 min and short calibration (concentration range between 1.0 × 10^−6^ and 1.0 × 10^−3^ M) were performed daily before measurements in order to test the sensor performance.

The TBZ is slightly soluble in water but can be soluble in acidic media; thus, the solution (*c* = 2.0 × 10^−3^ M) was prepared using HCl (*c* = 2.0 M) addition. pH in the TBZ solution was 2.6.

For the response measurements, TBZ (*c* = 2.0 × 10^−3^ and 5.0 × 10^−5^ M) was incrementally added to distilled water (*V* = 20 mL) using in-house software. Dynamic response measurements were performed by adding TBZ (*c* = 2.0 × 10^−3^ and 5.0 × 10^−4^ M) to distilled water (*V* = 50 mL) with 45 s pause between additions. Signal drift was measured in TBZ solution (*c* = 2.0 × 10^−3^ M, *V* = 20 mL). The influence of potential interferences (*V* = 20 mL, *c* = 1.0 × 10^−2^ M for all but fuberidazole and carbaryl (*c* = 2.5 × 10^−4^ M)) was investigated using the fixed interference method [29]. NaOH and HCl solutions (*c* = 1.0, 1.0 × 10^−1^ and 1.0 × 10^−2^ M) were used for measuring the influence of pH on the sensor response. For real samples preparation, chopped peel of bananas, oranges, lemons, clementines, and limes (*m* = 100 g) were placed in a glass and covered with distilled water (*V* = 185, 165, 195, 160, and 200 mL, respectively), followed by pH adjusting to 2.6. After 24 h, the samples were filtered through the gauze and measurements were performed (*V* = 15 mL).

## 3. Results and Discussion

### 3.1. Sensor Surface Morphology

**Figure 1:**
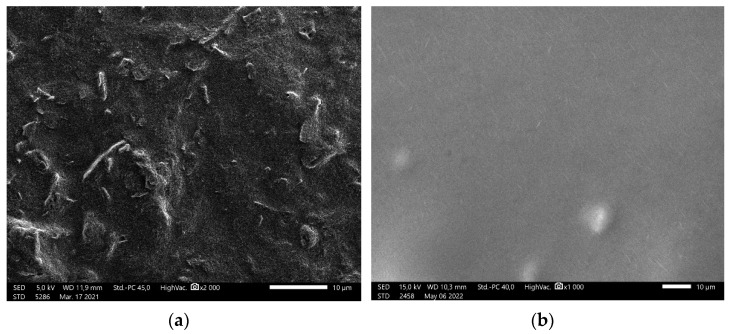
SEM images of sensor surface: (**a**) graphene modified; (**b**) PVC membrane.

The sensor surface morphology before and after modification was examined. The sensor surface after the drop-casting of graphene layer (Figure 1a) shows a modestly rough nonporous surface. Membrane surface, after modification with PVC membrane mixture on the graphene modified graphite core of the sensor (Figure 1b), is nonporous and smooth, with visible oriented carbon nanotubes. The orientation of carbon nanotubes occurs because of the surface tension changes during the drying process of the membrane production.

### 3.2. Response of the Sensor

The new MWCNT-OSO_3_^−^TBZ^+^ sensor is a solid-state electrode with a liquid membrane based on MWCNTs covalently connected with a sulfate group and TBZ ion as a sensing material. It exhibits a potentiometric response to TBZ according to the Nernst equation:

(1) $$E=E^{0}+S\times \log\;a_{TBZ^{+}}$$

where *E* is electrode potential, *E*^0^ is standard electrode potential, *S* is the slope of the sensor, and $a_{TBZ^{+}}$ is the activity of the TBZ cation. At 25 °C, the Nernstian slope for the TBZ cation amounts 59.2 mV/decade of activity.

The sensor material of the new MWCNT-OSO_3_^−^TBZ^+^ sensor dissociates as follows:

(2) $$\mathrm{MWCNT}-{\mathrm{OSO}}_{3}^{-}{\mathrm{TBZ}}^{+}⇄\mathrm{MWCNT}-{\mathrm{OSO}}_{3}^{-}+{\mathrm{TBZ}}^{+}$$

In the sensor membrane, the anionic component of the sensing material should be hydrophobic with a long alkyl chain to prevent high mobility. However, MWCNT-OSO_3_^−^ is not mobile in the membrane; thus, it represents a repulsive electrostatic barrier for penetration of anions. In addition, it is sensitive to changes in analyte solution, considering its good electrical properties. ${\mathrm{TBZ}}^{+}$ ions, present in the membrane near the MWCNT-OSO_3_^−^, regulate their charge, resulting in ion-to-electron transduction, which could be described as the asymmetric capacitor between the electrons in the MWCNT-OSO_3_^−^ wall and ${\mathrm{TBZ}}^{+}$ in the membrane. The concentration of TBZ in the solution will affect the electrical double layer on MWCNT-OSO_3_^−^ near the membrane/solution interface. It will cause ion-to-electron transduction and generation of an electromotive force that depends on the quantity of charge in the electrical double layer [30,31,32]; thus, it can be understood as the response mechanism of the new MWCNT-OSO_3_^−^TBZ^+^ sensor. The primary use of that sensor is TBZ determination using direct potentiometry.

**Figure 2:**
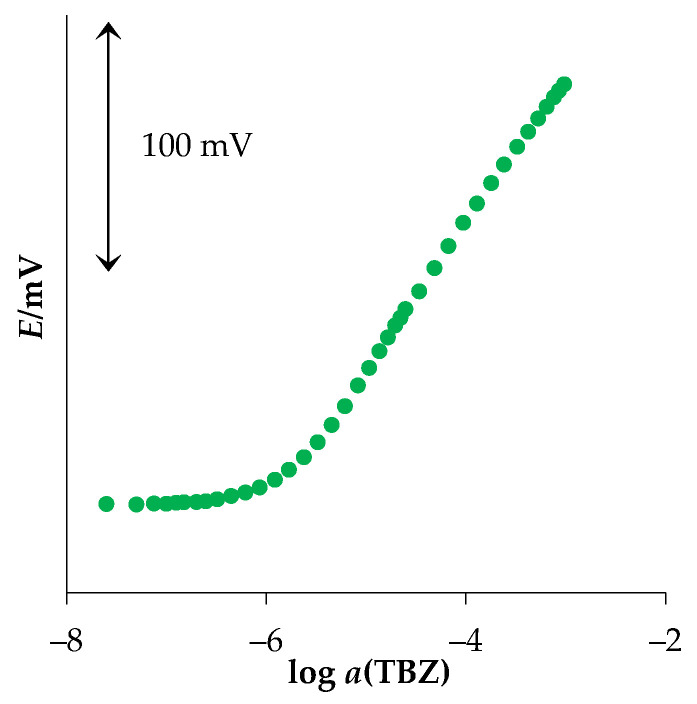
Response to thiabendazole (TBZ), obtained using the MWCNT-OSO_3_^−^TBZ^+^ sensor.

In response, characterization of the new MWCNT-OSO_3_^−^TBZ^+^ sensor, TBZ solution (*c* = 2.0 × 10^−3^ and 5.0 × 10^−5^ M) was used as the analyte. The investigated concentration range was 2.5 × 10^−8^ to 1.0 × 10^−3^ M. The measured response can be seen in Figure 2. Table 1 presents the corresponding data and statistics obtained using linear regression analysis. The activity coefficients and limit of detection were calculated using the Davies equation and IUPAC recommendations [33], respectively.

The new MWCNT-OSO_3_^−^TBZ^+^ sensor revealed a Nernstian response (60.4 mV/decade of activity) to TBZ in a concentration range between 8.6 × 10^−7^ and 1.0 × 10^−3^ M. Due to its wide concentration range, as well as its low limit of detection, it could be applicable for TBZ determination in various real samples.

The comparison of the new MWCNT-OSO_3_^−^TBZ^+^ sensor with other potentiometric sensors for TBZ determination is presented in Table 2. It can be seen that there are only two published scientific articles focused on potentiometric determination of TBZ. Compared with the sensors described, the new MWCNT-OSO_3_^−^TBZ^+^ sensor has significantly better analytical properties, such as lower limit of detection, wider measuring concentration range, faster response and prolonged lifetime.

**Figure 3:**
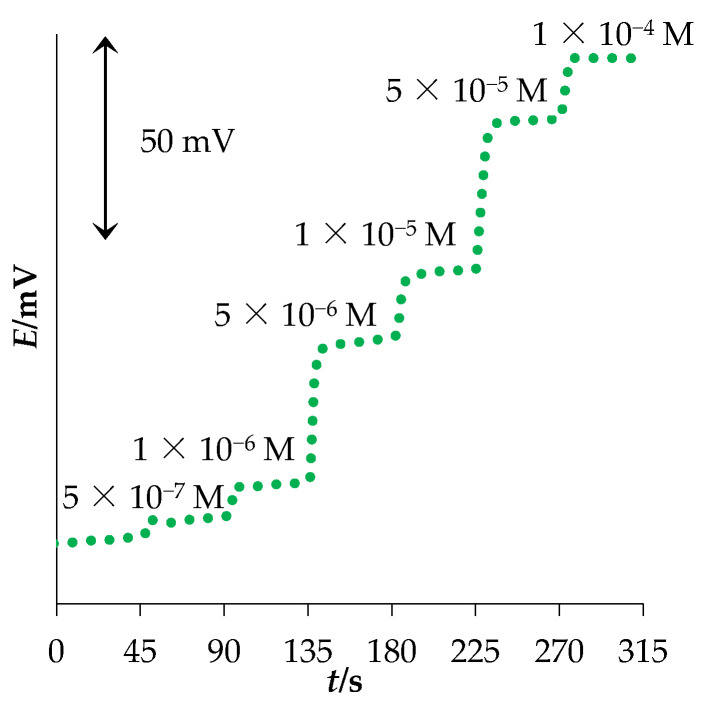
Dynamic response of the new MWCNT-OSO_3_^−^TBZ^+^ sensor.

Dynamic response time is another sensor characteristic that is also important for its applicability for TBZ determination. It represents the time needed to attain 90% of the final potential value after a sudden change in TBZ concentration [35]. The response time measurements were performed by changing TBZ concentration every 45 s, in the range between 5.0 × 10^−7^ and 1.0 × 10^−4^ M. The resulting response is presented in Figure 3. It can be seen that the new MWCNT-OSO_3_^−^TBZ^+^ sensor has a fast response, with an average response time of 8 s.

The stability of the sensor response can be observed using signal drift. It represents a change in the sensor potential as a function of time. Signal drift of the new MWCNT-OSO_3_^−^TBZ^+^ sensor was measured in TBZ solution (*c* = 2.0 × 10^−3^ M) using linear regression analysis, and it is described by the equation: *E* (mV) = −0.0007 × t(s) + 360.28. The calculated signal drift was −1.7 mV/h.

### 3.3. Determination of the Sensor Selectivity

The sensor’s selectivity is one of its most important properties that determine its applicability for TBZ determination in real samples. It can be estimated by calculating potentiometric selectivity coefficients ($K_{A,\;B}^{pot})$ that indicate how many times the interferent concentration can be higher than TBZ without changing the sensor potential measured for TBZ. For selectivity investigation, the fixed interference method [33] was used. The sensor response was measured in solution with fixed concentration of interferent (*c* = 1.0 × 10^−2^ or 2.5 × 10^−4^ M) and variable concentration of TBZ (*c* = 1.0 × 10^−6^ to 1.0 × 10^−3^ M or 2.5 × 10^−8^ to 2.5 × 10^−5^ M, respectively). The potentiometric selectivity coefficients were then calculated by mathematically adjusting the Nikolskii–Eisenman equation (Equation (3)) to experimental data using *Solver* (Microsoft Excel).

(3) $$E=E^{{}^{\circ}}+\frac{2303RT}{z_{A}F}\log\left[a_{A}+\overset{N}{\underset{B=1}{\sum }}K_{A,B}^{pot}a_{B}^{\frac{z_{A}}{z_{B}}}\right]$$

where $a_{A}$ and $z_{A}$ are activity and charge of the analyte ion, respectively, and $a_{B}$ and $z_{B}$ are activity and charge of the interfering ion, respectively. Table 3 presents calculated potentiometric selectivity coefficients of potential interferents that can be expected in real samples.

It can be concluded that the new MWCNT-OSO_3_^−^TBZ^+^ sensor is selective for TBZ; it can be used as a sensor for TBZ determination in real samples. $K_{A,\;B}^{pot}$ values were highest for carbaryl and fuberidazole, which was expected considering their chemical structure.

### 3.4. The pH Influence

**Figure 4:**
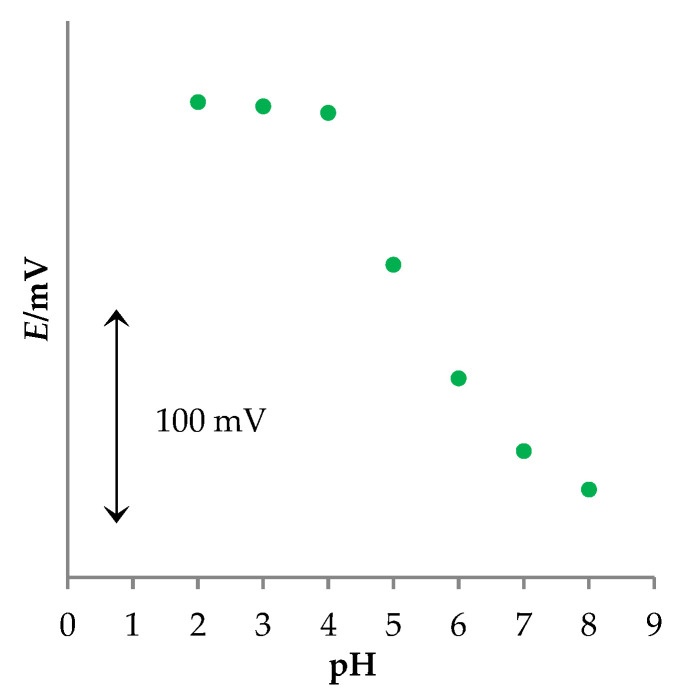
The influence of pH value on the potentiometric response of the new MWCNT-OSO_3_^−^TBZ^+^ sensor.

TBZ is slightly soluble in water. Its solubility increases in acidic media; thus, it was important to investigate the influence of pH on the sensor response. For that purpose, TBZ solution (*c* = 2.0 × 10^−^^3^ M) was used. The results are presented in Figure 4.

It can be observed that the sensor potential was stable in the pH range between 2 and 4, followed by a decrease in potential. Although the working pH range of the new MWCNT-OSO_3_^−^TBZ^+^ sensor is pretty narrow, it does not represent any limitation because TBZ can be dissolved only at low pH values (2.6).

### 3.5. TBZ Determination

The main purpose of the new MWCNT-OSO_3_^−^TBZ^+^ sensor is a TBZ determination using direct potentiometric measurements; thus, its applicability was tested in pure TBZ solutions and fruit peel samples.

#### 3.5.1. In Aqueous Samples

As aqueous samples, five TBZ solutions (*c* = 5.0 × 10^−4^, 1.0 × 10^−4^, 5.0 × 10^−5^, 1.0 × 10^−5^, and 3.0 × 10^−6^ M) were used. This investigation aimed to check the accuracy of the new MWCNT-OSO_3_^−^TBZ^+^ sensor for TBZ determination. After measuring the potential in the TBZ solution, its concentration was calculated using a calibration graph (response of the sensor) and the corresponding equation of a line. This procedure was followed by determining TBZ using the Gran method [36]. It was performed by adding 13 standard additions of TBZ (*c* = 2.0 × 10^−3^ M) in analyte solution (TBZ solution of known concentration) and by measuring the corresponding potential, which responds to TBZ according to Equation (4):

(4) E=k+Slogc

where *k* is constant, and *c* is the concentration of TBZ. After rearrangement, it follows:

(5) $$c=10^{\frac{E-k}{S}}=\frac{10^{\frac{E}{S}}}{10^{\frac{k}{S}}}$$

After every standard addition of TBZ, its concentration can be calculated as follows:

(6) $$c=\frac{c_{x}V_{0}+c_{s}V_{s}}{V_{0}+V_{s}}$$

where *c_x_* and *V*_0_ are the concentration and volume of the TBZ solution before standard addition, respectively. *c*_s_ and *V*_s_ are the concentration and volume of the standard addition solution, respectively. Combining Equations (5) and (6), and after rearrangement, it follows:

(7) $$10^{\frac{E}{S}}\left(V_{0}+V_{s}\right)=10^{\frac{k}{S}}c_{x}V_{0}+10^{\frac{k}{S}}c_{s}V_{s}$$

Dividing the sensor response with the standard addition with its response without addition and rearrangement leads to Equation (8):

(8) $$10^{\frac{E_{1}-E_{0}}{S}}\left(V_{0}+V_{s}\right)=V_{0}+\frac{1}{c_{x}}c_{s}V_{s}$$

**Figure 5:**
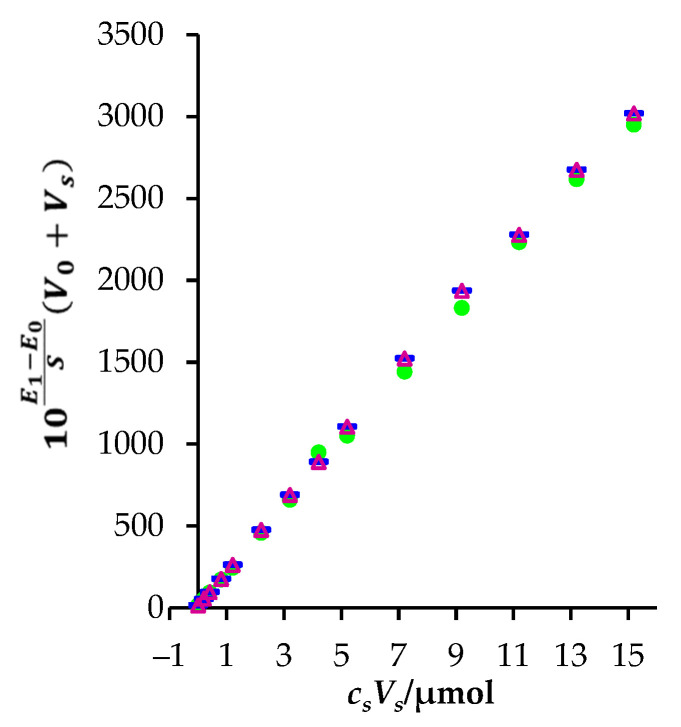
Gran plots obtained measuring TBZ concentration in aqueous sample of known concentration (*c* = 1.0 × 10^−5^ M, three repeated measurements).

After constructing a plot of $10^{\frac{E_{1}-E_{0}}{S}}\left(V_{0}+V_{s}\right)$ against $c_{s}V_{s}$, a linear graph is obtained. Its intercept is a negative value of the TBZ amount in the sample before standard additions. The concentration of TBZ in the analyte solution can be calculated by knowing the sample volume. Figure 5 presents the example of Gran plots obtained measuring TBZ concentration in aqueous samples of known concentration (*c* = 1.0 × 10^−5^ M, three repeated measurements).

Table 4 compares the results for TBZ determination in aqueous samples obtained using direct potentiometry and the Gran method.

Based on the results, it can be concluded that both direct potentiometry and the Gran method can be used for TBZ determination in real samples. Less accurate results, but still acceptable, were obtained in samples with lower TBZ concentration, which was expected considering that many factors can influence direct potentiometric measurements. The Gran method yields slightly more accurate results because it is based on multiple standard additions; thus, it was used to determine TBZ in oranges, lemons, bananas, clementines, and limes.

#### 3.5.2. In Real Samples

Peels of oranges, lemons, bananas, clementines, and limes were used as real samples. The fruit was purchased from the local store and peeled. Then, 100 g of fruit peel was chopped and covered with distilled water, followed by a pH adjustment to 2.6. After 24 h and filtration, determination of TBZ using the Gran method was performed. The accuracy of TBZ determination and potential influence of the matrix were investigated using the standard addition method, where 5 mL of TBZ (*c* = 2.0 × 10^−3^ M) was added to each fruit sample. The resulting data are presented in Table 5.

It can be seen that recoveries for all determinations are in the range ± 10%, which indicates the accuracy of the new MWCNT-OSO3^−^TBZ+ sensor and no influence of the matrix components on the TBZ determination in fruit samples.

### 3.6. The Lifetime of the Sensor

**Figure 6:**
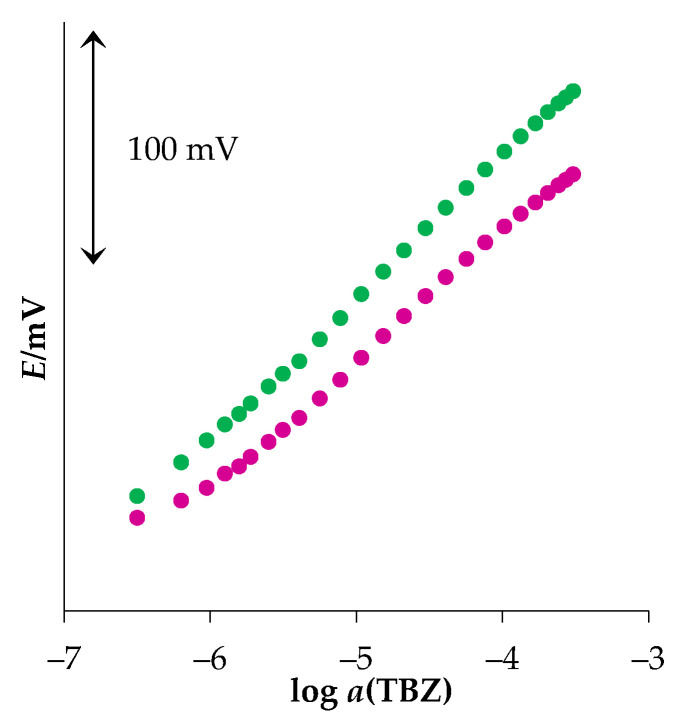
Responses of the new MWCNT-OSO_3^−^_TBZ^+^ sensor to TBZ in water (● new sensor, ● after approximately three months with daily measurements).

The lifetime of the sensor is the time in which the sensor can be used without changes in its performance. Every day before measurements, short calibration in a concentration range between 1.0 × 10^−6^ and 1.0 × 10^−3^ M was performed to check the performance of the new sensor and determine its lifetime. With daily use, it was approximately three months. After that period, the slope value of the new MWCNT-OSO_3^−^_TBZ^+^ sensor decreased by approximately 5%. Figure 6 presents typical responses of the MWCNT-OSO_3^−^_TBZ^+^ sensor to TBZ in water obtained using the new sensor and after approximately three months with daily measurements. After that time, the slope value decreased, the initial potential changed by approximately 10 mV, and the intercept value changed by approximately 55 mV. Although, the sensor could still be used for TBZ determination, its analytical performances have changed significantly. Between measurements, the sensor was stored in a moist atmosphere.

## 4. Conclusions

A new potentiometric solid-state MWCNT-OSO_3^−^_TBZ^+^ sensor for TBZ determination was prepared, containing a hybrid sensing material based on MWCNTs modified with a sulfate group and TBZ cation embedded in a liquid PVC membrane. The chemical modification of MWCNTs with TBZ cations improved the analytical performances of the sensor compared to the potentiometric sensor for TBZ determination published previously [22]. In addition, due to the new hybrid material and solid-state type of the sensor, good membrane stability, low signal drift, and reduced leaching of the sensing material were achieved, which prolonged the lifetime of the sensor. The new sensor obtained a Nernstian slope in a concentration range between 8.6 × 10^−7^ and 1.0 × 10^−3^ M. It was characterized by fast response time, good selectivity, and applicability for accurate TBZ determination in real samples (fruit peel).

## Figures and Tables

**Table 1:** Statistics of the response characteristics of the MWCNT-OSO_3_^−^TBZ^+^ sensor to TBZ ^1^.

Parameters	
Slope (mV/decade of activity)	60.4 ± 0.8
Standard error	1.6
Correl. coeff. (R^2^)	0.9991
Detection limit (M)	6.2 × 10^−7^
Useful conc. range (M)	8.6 × 10^−7^–1.0 × 10^−3^

^1^ Average of 5 determinations ± confidence limits (*p* = 0.95).

**Table 2:** The comparison of the performance of the new MWCNT-OSO_3_^−^TBZ^+^ sensor with the performance of other potentiometric sensors for TBZ determination.

Type of the Electrode	ISE with Liquid Membrane and Inner Electrolyte	ISE with Liquid Membrane and Inner Electrolyte	Solid-State ISE with Liquid Membrane
Ionophore	(TBZ)_3_(PMo_12_O_40_)_2_^1^	(TBZH_2_)_3_(PMo_12_O_40_)_2_^2^	MWCNT-OSO_3_^−^TBZ^+^
Slope [mV/decade of activity]	30	30	60.4 ± 0.8
Detection limit [M]	1.00 × 10^−5^	-	6.2 × 10^−7^
Useful conc. range [M]	1.0 × 10^−5^–1.0 × 10^−2^	1.0 × 10^−5^–1.0 × 10^−2^	8.6 × 10^−7^–1.0 × 10^−3^
Dynamic response [s]	40–50 (*c* = 10^−3^–10^−2^ M); 120–180 (*c* = 10^−6^–10^−4^ M)	40–50 (*c* = 10^−3^–10^−2^ M); 120–180 (low concentration)	8 (*c* = 5.0 × 10^−7^–1.0 × 10^−4^)
Working pH range	4–5	3–4	2–4
Lifetime [days]	65	60–65	90
Ref.	[22]	[34]	This work

^1^ Ion pair of TBZ with 12-molybdophosphoric acid. ^2^ Ion pair of TBZH_2_^2+^ with 12-molybdophosphoric acid.

**Table 3:** Potentiometric selectivity coefficients of the new MWCNT-OSO_3_^−^TBZ^+^ sensor obtained using the fixed interference method.

Interference	$K_{A,\;B}^{pot}$
Ammonium	5.60 × 10^−4^
Sodium	3.42 × 10^−4^
Calcium	7.20 × 10^−5^
Magnesium	1.60 × 10^−5^
Ascorbic acid	8.34 × 10^−4^
Citric acid	3.70 × 10^−3^
Potassium	5.42 × 10^−4^
Copper	3.59 × 10^−3^
Zinc	4.36 × 10^−4^
Lithium	2.05 × 10^−4^
Iron (III)	3.10 × 10^−3^
Iron (II)	2.34 × 10^−3^
Glucose	2.51 × 10^−4^
Fructose	2.21 × 10^−4^
Carbaryl	2.73 × 10^−2^
Fuberidazole	5.03 × 10^−2^

**Table 4:** Results of the TBZ determination in aqueous samples obtained using direct potentiometry and the Gran method ^1^.

TBZ Added (M)	TBZ Found (M) ± SD		Recovery (%)	
	Direct Potentiometry	Gran Method	Direct Potentiometry	Gran Method
5.00 × 10^−4^	4.74 × 10^−4^ ± 4.75 × 10^−6^	4.96 × 10^−4^ ± 5.30 × 10^−6^	94.8	99.2
1.00 × 10^−4^	1.08 × 10^−4^ ± 7.23 × 10^−6^	1.03 × 10^−4^ ± 1.45 × 10^−6^	108.0	103.0
5.00 × 10^−^^5^	5.18 × 10^−5^ ± 5.28 × 10^−7^	5.20 × 10^−5^ ± 8.06 × 10^−7^	103.6	104.0
1.00 × 10^−5^	1.05 × 10^−5^ ± 6.39 × 10^−7^	1.13 × 10^−5^ ± 4.88 × 10^−7^	105.0	113.0
5.00 × 10^−6^	4.09 × 10^−6^ ± 1.85 × 10^−7^	5.60 × 10^−6^ ± 2.36 × 10^−7^	81.8	112.0

^1^ Average of three determinations.

**Table 5:** Results of the TBZ determination in real samples obtained using Gran method ^1^.

Sample	TBZ Found (M) ± SD	TBZ Found (mg/g of the Peel)	Standard Addition Method		
			TBZ Added (mol)	TBZ Found (mol)	Recovery (%)
Orange	2.34 × 10^−4^ ± 1.65 × 10^−5^	0.078	1.00 × 10^−5^	9.51 × 10^−6^ ± 3.90 × 10^−7^	95.1
Lemon	3.29 × 10^−4^ ± 3.28 × 10^−5^	0.129	1.00 × 10^−5^	1.07 × 10^−5^ ± 5.73 × 10^−7^	107.0
Banana	2.39 × 10^−4^ ± 2.88 × 10^−5^	0.089	1.00 × 10^−5^	1.10 × 10^−5^ ± 2.54 × 10^−7^	110.0
Clementine	1.70 × 10^−4^ ± 8.98 × 10^−6^	0.055	1.00 × 10^−5^	1.03 × 10^−5^ ± 3.77 × 10^−7^	103.0
Lime	1.53 × 10^−4^ ± 7.52 × 10^−6^	0.062	1.00 × 10^−5^	1.05 × 10^−5^ ± 8.53 × 10^−7^	105.0

^1^ Average of three determinations.

## Data Availability

The data presented in this study are available on request.
